# Mortality associated with cardiovascular diseases in three hospitals of Lomé-city

**Published:** 2011-11-15

**Authors:** Findibe Damorou, Komlavi Yayehd, Soulemane Pessinaba, Tchaa Tcherou, Johnson Amonu

**Affiliations:** 1 Department of Cardiology, Campus University Teaching Hospital of Lomé, Togo

**Keywords:** Mortality rate, cardiovascular diseases, Cerebrovascular stroke, Togo

## Abstract

The aims of present study were; to determine the mortality rate related to cardiovascular diseases and the causes of those deaths in local hospitals. We conducted a cross sectional study carried out from January 2005 to June 2006, in three hospitals of Lomé. All deaths registered in the departments of cardiology and neurology from cardiovascular diseases were taken into account. The number of patients who died from cardiovascular diseases was 200 on 2386 admitted patients (11.86%). The average age was 54.4±15.6 years (range: 18-102). Cerebrovascular stroke was the most represented disease (56.9%), followed by heart failure (35.7%), and cardiac arrhythmias (15.9%). Hospital mortality due to cardiovascular afflictions was high in Lome-city due to the deficient organization of the cardiovascular resuscitation units. Better organization, more equipment for cardiovascular care units and better public health efforts surrounding control of cardiovascular risk factors could go a long way towards the reduction of this high mortality rate of cardiovascular diseases in our hospitals.

## Introduction

In most industrialized countries, the last two decades have been characterized by a further significant reduction in mortality. Summary measures of mortality, such as the age-standardized death rate, have declined in parallel with reductions in cardiovascular diseases mortality [1]. Cardiovascular diseases represent 30% of global mortality and are therefore a major public health issue [2]. They are the main cause of death in Europe, accounting for 49% of all deaths (and 30% of all premature deaths before the age of 65) [3]. Although age-specific mortality rates from cardiovascular diseases have halved in Western Europe in the last 20 years, the prevalence of cardiovascular disease is actually increasing due to an ageing population [3]. In sub-Saharan Africa, the nature and the rates of these cardiovascular diseases are still inadequately known because of the very recent organization of cardiovascular emergency care units and lack of studies going this way [4]. The aim of the present study is to determine the hospital mortality rate due to cardiovascular afflictions and point out the causes of those deaths.

## Methods

Three hospitals in Lome were used as settings for this study, namely the departments of cardiology and neurology of the Tokoin university teaching hospital (first national reference centre), the Campus university teaching hospital (second national reference centre) and Bê District hospital. None of these centers has cardiovascular resuscitation units. It was a cross sectional study on 151 medical files gathered from January 2005 to June 2006. We at the outset consulted registers in which were mentioned deaths; there-after, we preceded with research of corresponding files in record books. We have included all the files of patients admitted and died from cardiovascular causes.

### Data analysis

All quantitative parameters are presented as the average ± mean deviation, and all qualitative parameters are presented as the number and its corresponding percentage. Parameters studied for every file were: age, sex and causes of deaths. Data analysis was performed using the software SPSS (SPSS for windows; 2005). The ANOVA parametric test was used for means comparison with a significant p value inferior to 5%.

## Results

### Mortality

We noticed 200 deaths over 1685 patients admitted for cardiovascular afflictions; this represented a hospital mortality rate of 11.86%. The study was done on 151 files. The 49 remaining were not founded in records. Cardiovascular deaths registered in the Campus teaching hospital are available in Table 1.

**Table 1:** Cardiovascular deaths registered in 3 hospitals in Lomé town from January 2005 to June 2006

	Number of death	Number of patients admitted	Files found
Tokoin UTH*	72	785	48
Campus UTH*	117	799	91
Bê District Hospital	11	101	11
Total	200	1685	151

^*^University Teaching Hospital

### Age and sex

There were 83 females and 68 males; the sex-ratio was 0.82. The average age was 54.4 ± 15.6 (range: 28-102 years); 53.9 ± 19.4 (range: 18-98 years) in males and 55.9 ± 16.4 (33-102 years) in females (p = 0.49). The most represented age group was 30 to 69 years (Table 2).

**Table 2:** Distribution of deaths according to age groups in 3 hospitals in Lomé town from January 2005 to June 2006

Age group	Number	Percentage (%)
18 – 29 years	6	3.97
30 – 69 years	107	70.86
> 70 years	38	25.17
Total	151	100

### Causes of deaths

Most of patients died from cerebrovascular stroke and heart failure (Table 3).

**Table 3:** Distribution of deaths according to the causes in 3 hospitals in Lomé town from January 2005 to June 2006

Causes of deaths	Number	Percentage (%)
Cerebrovascular Stroke	70	46.36
Heart Failure	54	35.76
Cardiac arrhythmia	24	15.89
Myocardial infarction	3	1.99
Total	151	100

## Discussion

This study is the first of this nature carried out on global mortality of cardiovascular afflictions in some Togolese hospitals. It was carried out in specialized medical departments so as to collect reliable and precise data. The mortality rate was 11.86%. It was especially high compared to that of industrialized countries. In the European Union (27 countries), coronary heart diseases mortality in men declined from 139/100,000 in 1985-1989 to 93/100,000 in 2000-2004 (-33%). In women, this rate fell from 61/100,000 to 44/100,000 (-27%). In those countries, a decline of more than 30% was also registered in cerebrovascular diseases mortality for both sexes. In the Russian Federation and other countries of the former Soviet Union, coronary heart diseases rates in 2000-2004 were exceedingly high, around 380/100,000 men and 170/100,000 women in Russia, 430 for men and 240 for women in Ukraine, 420 and 200 in Belarus. For cerebrovascular diseases, a similar situation was registered, with mortality rates of 226/100,000 for men and 159/100,000 for women in 2004 in the Russian Federation, and more than 24% increase since the late 1980s for men and 15% for whe late 1980s for men and 15% for women. Coronary heart diseases and cerebrovascular diseases mortality continued to decline in most Latin American countries, Australia and other areas considered, including Asia (even if with marked differences) [5]. The difference with the European rates is probably due to the lack of adequate structures for cardiovascular resuscitation in public hospitals of Lomé.

In this study, deaths due to cardiovascular diseases concerned males as well as females. Women's death proportion (55.62%) was slightly above that of men's (44.37%): sex-ratio of 0.79. In public hospitals of Lomé, cerebrovascular strokes caused 46.36% of deaths, followed by heart failure (35.76%) then cardiac arrhythmias (15.89%). We have reported in a former study on cardiovascular emergencies in Campus university teaching hospital [4] that cerebrovascular strokes were the most frequent cause of death with a lethality rate of 85.6%. This confirms in one way the delay in the consultation of hypertensive patients and in other way the difficulties in the management of this disease in our hospitals.

## Conclusion

Hospital mortality due to cardiovascular diseases is very high in Lomé. Better organization and more equipment for cardiovascular intensive care units will be essential and better public health efforts surrounding control of cardiovascular risk factors could go a long way to reduce this high mortality rate of cardiovascular diseases in our hospitals. Large national studies have to be carried out in order to specify all the data in relation to the morbidity and mortality by cardiovascular diseases in Togo.
